# Patient safety culture in times of the COVID-19 pandemic: a cross-sectional study in a hospital

**DOI:** 10.1590/0034-7167-2023-0187

**Published:** 2024-08-30

**Authors:** Amanda Lobato Lopes, Rosana Aparecida Pereira, Laura Martins Valdevite Pereira, Felippe Micheli Costa de Castilho, Fernanda Raphael Escobar Gimenes

**Affiliations:** I Universidade de São Paulo. Ribeirão Preto, São Paulo, Brazil

**Keywords:** Patient Safety, Quality of Healthcare, Organizational Culture, Hospital, COVID-19, Seguridad del Paciente, Calidad de la Atención de Salud, Cultura Organizacional, Hospital, COVID-19

## Abstract

**Objectives:**

to assess patient safety culture during the COVID-19 pandemic and identify the dimensions that need to be improved in hospital settings and which sector, open or closed, direct or indirect care, exhibits a higher level of safety culture.

**Methods:**

a descriptive and cross-sectional study. The validated version for Brazil of the Hospital Survey on Patient Safety Culture instrument was applied to assess patient safety culture. Those dimensions with 75% positive responses were considered strengthened.

**Results:**

all dimensions presented results lower than 75% of positive responses. Closed sectors showed a stronger safety culture compared to open ones. Indirect care sectors had a low general perception of patient safety when compared to direct care sectors.

**Conclusions:**

with the pandemic, points of weakness became even more evident, requiring attention and incisive interventions from the institution’s leaders.

## RESUMEN

**Objetivos:**

evaluar la cultura de seguridad del paciente en el contexto de la pandemia COVID-19 e identificar las dimensiones que necesitan ser mejoradas en el ambiente hospitalario y qué sector, abierto o cerrado, de asistencia directa o indirecta, exhibe un mayor nivel de cultura de seguridad.

**Métodos:**

estudio descriptivo y transversal. Para evaluar la cultura de seguridad del paciente se aplicó la version validada para Brasil del instrumento Hospital Survey on Patient Safety Culture. Se consideraron fortalecidas aquellas dimensiones con un 75% de respuestas positivas.

**Resultados:**

todas las dimensiones presentaron resultados inferiores al 75% de respuestas positivas. Los sectores cerrados mostraron una cultura de seguridad más fuerte en comparación con los abiertos. Los sectores de atención indirecta tenían una percepción general baja de la seguridad del paciente en comparación con los sectores de atención directa.

**Conclusiones:**

con la pandemia, los puntos de debilidad se hicieron aún más evidentes, requiriendo atención e intervenciones incisivas por parte de los líderes de la institución.

## INTRODUCTION

On December 31, 2019, the Wuhan Municipal Health Commission reported on its website the occurrence of cases of atypical pneumonia in the People’s Republic of China^(1)^. On January 9, 2020, Chinese authorities determined that the disease outbreak was caused by a new coronavirus, SARS-CoV-2, publicly known as COVID-19, an infection responsible for a potentially fatal respiratory syndrome^(1)^.

Due to the rapid spread of the disease to several countries, on February 11, 2020, the World Health Organization (WHO) characterized the outbreak of the disease as a pandemic^(2)^. Between January 1, 2020 and May 18, 2021, 163,312,429 cases and 3,386,825 deaths were confirmed worldwide^(3)^. In Brazil, the first case of the disease occurred in the city of São Paulo on February 26, 2020^(4)^. In the city of Ribeirão Preto, state of São Paulo, in 2020, 41,977 cases and 1,045 deaths were confirmed^(5)^; in 2021, there were 73,272 cases and 1,995 deaths; and from January 1 to August 16, 2022, there were 53,402 cases and 368 deaths^(6)^.

Managing a pandemic requires a robust hospital structure that provides quick and assertive decision-making to control and spread the virus^(7)^. From this perspective, the COVID-19 pandemic challenged Healthcare Systems (HCS) on a global scale, and, in Brazil, this scenario required the Brazilian Health System (SUS – *Sistema Único de Saúde*) to also adapt to face the crisis^(8)^.

Faced with this adverse scenario, rapid changes in care delivery models were necessary, including increased workload, redeployment of personnel to unfamiliar clinical environments and the need to treat patients with a new and still little-known disease. Furthermore, healthcare professionals were encouraged to develop the ability to deal with the disease, adapting quickly to changes, overcoming challenges and resisting pressure^(9)^.

Previous research revealed that changes in organizational routines, scarcity of qualified human resources, fatigue of healthcare teams and overworked institutions under extreme pressure are factors that contribute to unsafe care, in addition to negatively affecting job satisfaction^(10)^. Therefore, investigating the impact of such factors on patient safety, especially in times of health crisis, is necessary.

Patient safety was defined by the WHO (2021)^(11)^ as “a framework of organized activities that creates cultures, processes, procedures, behaviours, technologies and environments in healthcare that consistently and sustainably lower risks, reduce the occurrence of avoidable harm, make error less likely and reduce its impact when it does occur”.

The WHO also highlighted the need to create high-reliability systems to ensure people’s access to quality and safe health services, through the development and maintenance of a transparent safety culture that promotes continuous learning and is not punitive^(11)^.

The term “safety culture” was used for the first time in the literature by the International Consultative Group on Nuclear Safety in its report on the Chernobyl accident, which occurred in 1986^(12)^. Since then, the term has been used by several institutions considered high risk, such as hospitals^(13)^. It is defined as the product of group and individual values, attitudes, perceptions and skills, which determine a pattern of behavior and commitment to safety management^(12)^.

In healthcare institutions, safety culture is defined as the association of individual and group actions that aim to reduce the occurrence of harm to patients as a result of interactions, attitudes and perceptions about safety issues. Studies have shown that there is a direct association between the implementation of a safety culture in healthcare institutions and a reduction in serious and fatal adverse events^(14-15)^.

Several factors interfere with health service safety culture, including failures in communication between teams, high workloads, the existence of a punitive culture and leadership hierarchization^(10,16-17)^. Therefore, in order to obtain information about the state of patient safety in a healthcare institution, investigations aimed at assessing culture are essential.

There are several instruments available in the literature aimed at assessing safety culture. In the present study, the instrument developed by the Agency for Healthcare Research and Quality (AHRQ) called Hospital Survey on Patient Safety Culture (HSOPSC) ^(18)^ was used, which was translated and validated for Brazilian culture^(19)^. The instrument aims to assess the several patient safety culture dimensions in the health service.

The HSOPSC was used in several international studies^(20-24)^. In an investigation conducted in three public hospitals in Kuwait, a HSOPSC psychometric assessment was carried out. Of the 22 items related to safety climate, all presented strong loadings between the factors (0.42-0.86). Furthermore, in relation to reliability analysis, the results were satisfactory (α> 0.60)^(22)^.

Brazilian researchers carried out a systematic review of the PubMed, Web of Science and Scopus databases with the aim of identifying studies that used the HSOPSC to collect data on safety culture and its contribution to improving quality and safety in hospital care^(25)^. A total of 33 studies were selected, conducted in 21 countries. As for teamwork dimensions, the results showed a strong load, and the dimensions with a weak load were related to punishment for errors related to patient care. They concluded that organizational culture was weak in the institutions assessed, negatively impacting patient safety and health outcomes^(25)^.

HSOPSC is self-administered and assesses 12 patient safety culture dimensions, two of which are related to patient safety results (1 - Frequency of events reported; and 2 - Overall perceptions of patient safety) and ten are related to safety culture (1 - Supervisor/manager expectations and actions promoting patient safety; 2 - Organizational learning—continuous improvement; 3 - Teamwork within units; 4 - Communication openness; 5 - Feedback and communication about error; 6 - Nonpunitive response to error; 7 - Staffing; 8 - Management support for patient safety; 9 – Teamwork across units; and 10 - Handoffs and transitions)^(19)^.

The score is calculated using a five-point Likert scale, assigning a numerical value to the answers given by study participants. The answers have categories that vary from 1 – agreement (totally disagree; disagree; neither agree nor disagree; agree; and totally agree) to 2 – frequency (never; almost never; sometimes; almost always; and always).

Based on the above, this study was designed to answer the following questions: what is the perception of hospital professionals about patient safety culture in times of the COVID-19 pandemic? Which dimensions of patient safety culture need to be improved? Which type of sector (open and closed) and type of care (direct and indirect) have the strongest patient safety culture?

## OBJECTIVES

To assess patient safety culture during the COVID-19 pandemic and identify the dimensions that need to be improved in hospital settings and which sector, open or closed, as well as those that offer direct and indirect care, exhibits a higher level safety culture.

## METHODS

### Ethical aspects

The study was approved by the *Universidade de São Paulo Escola de Enfermagem de Ribeirão Preto* Research Ethics Committee (REC), in accordance with Brazilian National Health Council Resolution 466/2012 of the Ministry of Health Brazilian National Research Ethics Council, which addresses ethics in research with human beings. The Informed Consent Form (ICF) was sent via email to study participants, who were informed that the research results would be intended for publication and that confidentiality and anonymity would be guaranteed.

### Study design, period and place

This is a descriptive and cross-sectional study, with a quantitative approach. The scientific writing checklist called STrengthening the Reporting of OBservational studies in Epidemiology (STROBE) was used^(26)^. The study was carried out in a large tertiary hospital, a highly complex center in the countryside of the state of São Paulo. The hospital is made up of three units, namely: campus; emergency unit; and children’s hospital. Data was collected between November 30, 2021 and September 30, 2022 in all sectors of the three units.

### Study population

The study’s target population was made up of all high school and higher education administrative professionals, high school and higher education healthcare professionals and other high school and higher education professionals. The sample adopted in this study was simple random sampling.

### Inclusion and exclusion criteria

Professionals who had been working at the hospital for more than three months and who worked at least 20 hours a week were included. Professionals who, during data collection, were on vacation or leave and occasional professionals without an employment relationship with the hospital, such as healthcare professions interns, academics and residents, were excluded. Forms that were not completely filled out were also excluded.

### Study protocol

#### Data collection

Data were collected through HSOPSC application. To ensure the quality and reliability of the data collected, AHRQ^(27)^ guidelines were used, with validation and inclusion of all professionals working in hospitals.

In addition, information was collected about the research participants, including gender, education level, position/function, hospital job tenure, weekly workload and direct interaction with patients.

Data collection was carried out in a mixed manner: (1) by providing the access link to the electronic instrument, which was prepared on the RedCap virtual platform; and (2) in person, with the form printed and distributed to study participants working in the three hospital units.

The link was made available from November 30, 2021 to February 7, 2022, sent by institutional email, with assistance from the Risk Management Service. However, due to the low participation of the target audience, collection began to be carried out in person through handing over printed forms from February 21, 2022 to September 30 of the same year. It is noteworthy that this change was previously authorized by the institution’s management. All floors of the three hospital units were visited by the researcher, who made prior contact with the person in charge of the sector. Those who agreed to participate in the research were sent the ICF via email and given a period of one week to complete it. Subsequently, the printed instruments were collected by the researcher, and the data was entered into the RedCap system.

#### Analysis of results, and statistics

Data were transferred from the RedCap platform to Microsoft Excel® 2016 spreadsheets. All analyzes were carried out in the R program (R Core Team, 2021) version 4.1.2, and the percentages of positive responses regarding patient safety culture dimensions were calculated. Alternatives 4 or 5 (agree/totally agree or almost always/always) were considered positive responses in positively formulated questions and alternatives 1 or 2 (totally disagree/disagree or never/rarely) in negatively formulated questions. Regarding negative responses, alternatives 1 or 2 were considered for positively formulated questions and 3 or 4 for negatively formulated questions. Responses recorded as “neither disagree nor agree” or “sometimes”, as proposed by AHQR, were considered neutral^(27)^.

Patient safety culture dimensions were considered strong areas, which obtained 75% positive responses. Dimensions that obtained 50% or less positive responses were considered fragile areas in need of improvement^(27)^.

The comparison between the open and closed sectors was carried out according to the classification proposed by Bianchi^(28)^, taking into account the flow of patients and family members. Open sectors were outpatient clinics, oncology sector, Liver Transplant Unit, Kidney Transplant Unit, in addition to psychiatry wards, medical clinic, hematology, gynecology and obstetrics, surgery, orthopedics, ophthalmology, otorhinolaryngology and pediatrics. Closed sectors were administration, accounting, human resources, Internal Regulation Center (IRC), Integrated Quality Center, nutrition and dietetics, distribution service, materials center, respiratory unit, microbiology laboratories, Rehabilitation Center (REC), dialysis unit, blood bank, transfusion agency, risk control, pharmacy, surgical center, post-anesthesia recovery, adult, pediatric and neonatal Critical Care Unit (CCU), neurology, Coronary Care Unit (CoCU), burns unit, chemotherapy center, Infectious Diseases Treatment Unit (IDTU), semi-intensive medical clinic and Epilepsy Surgery Center (CIREP).

A comparison was also made between direct and indirect care sectors, following Bartolomei’s^(29)^ classification as a theoretical framework, which distinguishes healthcare as direct, with care being offered in a direct relationship with users, and as indirect, being acts that provide physical and material comfort and safety to patients. For indirect care, all professionals working in management and administration, material distribution, pharmacy, nutrition and laboratory sectors were considered. Professionals from all outpatient clinics, wards, CCU, surgical center, treatment units, such as dialysis, burns, rehabilitation and others, were considered direct care. The Kruskal-Wallis non-parametric test was used, an appropriate choice for comparing three or more independent samples. The significance level adopted in this study was set at 95%.

## RESULTS

A total of 459 forms were completed, of which 111 (24.2%) were completed online, while 348 (75.8%) were completed in printed format. Regarding professionals’ work unit, 327 (71.2%) worked in the campus unit; 89 (19.4%) worked in the emergency unit; and 43 (9.4%) worked at the children’s hospital. Regarding the type of sector, there were 139 (30.3%) participants from open units and 320 (69.7%) from closed units. Considering the type of care, 388 (84.5%) provided direct patient care, and 71 (15.5%) provided indirect care. Among participants, the majority were female (367; 80.1%) and with a mean age of 43.53 years (21.07 ± 70.22). Table 1 presents participant characteristics.

**Table 1:** Characterization of professionals working in the three hospital units participating in the research (N=459), Ribeirão Preto, São Paulo, Brazil, 2022

Variables	Frequency n (%)
Sex	
Female	367 (80.1)
Male	91 (19.9)
Education	
High school or less	134 (29.2)
Higher education	142 (31)
Specialization	115 (25.1)
Master’s or doctoral degree	68 (14.8)
Position/function	
Assistants/technicians*	248 (54.0)
Graduates**	211 (46.0)
Hospital job tenure	
≤ 5 years	161 (35.1)
6 to 10 years	86 (18.7)
11 to 15 years	53 (11.5)
16 to 20 years	27 (5.9)
>20 years	132 (28.8)
Weekly workload	
< 40 hours	264 (57.5)
≥ 40 hours	295 (42.5)
Direct contact with patient	
Yes	336 (73.2)
No	123 (26.8)

* Assistants/techniäans: nursing assistant and technician; other technical level professionals, such as those working in electrocardiogram, laboratory, radiology and pharmacy; administrative assistant/secretary; other; **Graduates: clinical staff physician/assistant physician; nurse; pharmacist/biochemist/biologist/biomedical; nutritionist;physiotherapist, respiratory therapist, occupational therapist or speech therapist; psychologist; social worker; administration/direction.

**Figure 1:**
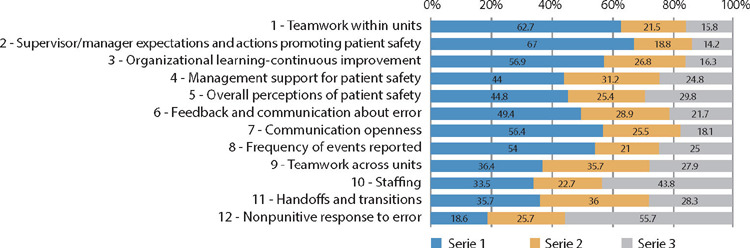
Distribution of negative, neutral and positive responses to the 12 safety dimensions assessed through the Hospital Survey on Patient Safety Culture, Ribeirão Preto, São Paulo, Brazil, 2022

Considering the 42 HSOPSC questions, which were grouped into 12 dimensions of safety culture, the percentage of positive, neutral and negative responses was calculated, as shown in Figure 1.

None of the 12 dimensions assessed reached 75% of positive responses. Despite this, the dimensions that expressed the highest percentages of positive responses stand out, i.e., 67% in “Supervisor/manager expectations and actions promoting patient safety” and 62.7% in “Teamwork within units”.

Furthermore, seven of the 12 dimensions obtained 50% or less positive responses, representing weak areas of patient safety that require improvement, in descending order: “Feedback and communication about error” (49,4%); “Overall perceptions of patient safety” (44,8%); “Management support for patient safety” (44%); “Teamwork across units” (36,4%); and “Handoffs and transitions” (35,7%). The two dimensions with the lowest percentages of positive responses stood out: “Staffing” (33.5%); and “Nonpunitive response to error” (18.6%).

**Figure 2:**
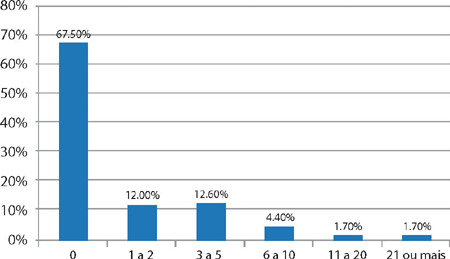
Adverse events reported by professionals in the last 12 months (N = 459), Ribeirão Preto, São Paulo, Brazil, 2022

Regarding the variable “adverse events reported by professionals in the last 12 months”, Figure 2 showed that the majority (67.5%) did not report anything.

With regard to the general level of patient safety, the results revealed that, of the 459 (100%) participants, 218 (47.5%) considered the hospital’s patient safety to be very good.

In the present study, the percentage of positive responses for patient safety was compared between closed and open sectors. Table 2 shows that closed sectors obtained higher percentages of positive responses in all 12 patient safety dimensions compared to open sectors; however, the results were not significant, except for “Supervisor/manager expectations and actions promoting patient safety” (p-0.015), “Feedback and communication about error” (p-0.0077), “Communication openness” (p-0.0156) and “Teamwork across units” (p-0.0039).

**Table 2:** Distribution of positive responses to the 12 dimensions of patient safety assessed through the Hospital Survey on Patient Safety Culture, ac cording to the type of sector, Ribeirão Preto, São Paulo, Brazil, 2022

Patient safety dimensions	Type of sector (%)		*p* value*
	Open	Closed	
1 - Teamwork within units	62.2	62.7	0.7481
2 - Supervisor/manager expectations and actions promoting patient safety	65.7	67.5	0.015
3 - Organizational learning—continuous improvement	51.1	59.5	0.0996
4 - Management support for patient safety	40	45.7	0.1464
5 - Overall perceptions of patient safety	41.4	46.3	0.5897
6 - Feedback and communication about error	48.6	49.1	0.0077
7 - Communication openness	55.6	55.7	0.0156
8 - Frequency of events reported	53	54.4	0.6029
9 - Teamwork across units	31.9	39.2	0.0039
10 - Staffing	28.4	35.8	0.3583
11 - Handoffs and transitions	34.2	36.4	0.843
12 - Nonpunitive response to error	14.6	20.3	0.4166

* *Kruskal-Wallis.*

A comparison of patient safety culture was also carried out between sectors that provide direct patient care and sectors that provide indirect care. Table 3 reveals that, in sectors where indirect care is provided to patients, the percentage of positive responses was higher (eight of the 12 dimensions) than when compared to sectors that provide direct care. However, the results were not significant, except for “Organizational learning—continuous improvement”, with a final value measured at p=0.0033.

**Table 3:** Distribution of positive responses to the 12 safety dimensions assessed through the Hospital Survey on Patient Safety Culture according to the type of care provided to patients, Ribeirão Preto, São Paulo, Brazil, 2022

Patient safety dimensions	Tipo de Care (%)		*p* value*
	Direct	Indirect	
1 - Teamwork within units	62.6	62.3	0.5256
2 - Supervisor/manager expectations and actions promoting patient safety	65.7	74	0.2086
3 - Organizational learning—continuous improvement	56	62	0.0033
4 - Management support for patient safety	42.3	53.5	0.497
5 - Overall perceptions of patient safety	45.2	43	0.1183
6 - Feedback and communication about error	48.6	58.3	0.8998
7 - Communication openness	55.7	63.8	0.7259
8 - Frequency of events reported	53.9	54.9	0.8569
9 - Teamwork across units	37.5	47.5	0.074
10 - Staffing	34	31.1	0.0589
11 - Handoffs and transitions	35.8	33.8	0.3574
12 - Nonpunitive response to error	18.2	20.7	0.4737

* *Kruskal-Wallis.*

## DISCUSSION

The general objective of this study was to assess patient safety culture during the COVID-19 pandemic and identify the dimensions that need to be improved in the hospital. The results showed that none of the 12 safety dimensions assessed through the HSOPSC were considered strong areas, as they did not present a percentage of ≥75% of positive responses.

This same setting was presented in a scoping review that brought together several studies developed in Brazil. According to the researchers, the majority of Brazilian hospitals do not have any strong dimension^(30)^.

However, in the present study, 47.5% of participants consider the hospital’s patient safety to be “very good”. Some dimensions had the potential to be strengthened, such as “Supervisor/manager expectations and actions promoting patient safety” and “Teamwork within units”. This result corroborates the results of research conducted in a philanthropic hospital in Diamantina, state of Minas Gerais^(31)^. The results demonstrate the importance of leaders expressing interest in the topic, encouraging good practices, investing in safe work processes and praising actions performed with excellence, thus motivating employees to improve patient safety. When professionals achieve this vision, teamwork becomes a reality, as this research illustrates, in which support and respect between people are present, thus favoring safe and quality care.

In a systematic review carried out in 21 countries, mostly European and Asian^(25)^, it was demonstrated that even considering the diversity of each one, the dimensions that appear highlighted with the lowest positive response scores were “Nonpunitive response to error” and “Staffing”, representing the most fragile areas as well as in the present study, which showed percentages of 18.6% and 33.5%, respectively.

In the context of patient safety, “Nonpunitive response to error” exposes the culture of blame that is still present in many hospital institutions, where professionals feel afraid to report errors due to the possible fear of being held responsible and, consequently, punished, leading to underreporting of events^(32)^. This point can be confirmed by the percentage of reports in the 12 months prior to the survey, in which more than half (67.5%) of professionals did not report. Other studies that used the same instrument obtained similar results for these dimensions^(30,32-33)^.

This situation of underreporting of adverse events is aggravated by the “Staffing” dimension, highlighted as the second most fragile area of the study. Adding to the adverse scenario of the COVID-19 pandemic, it brings an increase in workload, redistribution of personnel to unfamiliar clinical environments and the need to treat patients with a new disease, resulting in increased professional stress^(9)^, factors that may result in underreporting.

When relating the fragile areas of the hospital investigated during the pandemic to previous studies conducted in other scenarios and contexts, it appears that the pandemic highlighted points of fragility that were already considered challenging, including the culture of blaming and punishing errors, expressed in these studies by the lower percentage of positive responses for “Nonpunitive response to error” and “Staffing”, related to work overload and inadequacy of human resources^(30,32-33)^.

In the present study, open sectors presented a lower percentage of positive responses in all dimensions of patient safety when compared to closed sectors. As no previous studies were found that assess the dimensions between open and closed sectors, this comparison was not possible.

With regard to direct and indirect care sectors, indirect care sectors, which predominantly include management and administration, obtained a higher percentage of positive responses for most dimensions. A study carried out in the USA as an extension of HSOPSC showed that management sectors tend to believe that patient safety culture in the institution is stronger than it actually is^(34)^. This fact, in the present study, may have influenced the greater positivity of patient safety dimensions in indirect sectors.

On the other hand, in “Overall perceptions of patient safety”, indirect care sectors had a lower positive response percentage (43%) than direct care sectors (45.2%), highlighting possible difficulty in assessing safety culture in the institution. Related to this context, one of the main challenges of data collection in the present study stands out, which would be the low engagement of professionals in sectors with indirect care, especially administrative professionals. According to reports from these professionals, they do not understand their role in patient safety because they are not directly linked to care. Still in accordance with the study carried out in the USA, administrative positions - indirect care - are areas with greater gaps in patient safety culture perception, reinforcing the importance of training these professionals on the subject so that they become co-responsible for patient safety and the institution as a whole achieves a shared safety culture^(34)^.

Furthermore, research carried out in the Intensive Care Unit of a university hospital showed that the group of patients who presented safety incidents related to administrative failures required longer hospitalization and greater care by the nursing team^(35)^. This scenario reveals that all professional areas involved in the care process, whether direct or indirect, must participate and engage in safety strategies. Bearing this in mind, it is important that institutions rethink the current safety culture, in order to become a high reliability organization. Such organizations present a condition of full and persistent attention aimed at early recognition of errors and immediate intervention, in order to prevent such events from becoming catastrophic. Furthermore, high-reliability organizations cultivate resilience by relentlessly prioritizing safety over other performance measures^(36)^.

### Study limitations

Among the limitations of this study, it is possible to mention the lack of research using the same instrument, carried out during the COVID-19 pandemic, making it impossible to compare the results. Furthermore, there was a lack of adherence by professionals to participate in the research and/or inadequate completion of the form.

Another limitation is the possibility of response bias by participants. Depending on individual sensitivity or perceptions, participants may be inclined to provide responses that reflect a more favorable view of safety culture, which may affect the accuracy of results. Furthermore, the simple random sample may not completely represent the diversity of hospital contexts, limiting the generalization of results to other health institutions.

### Contributions to nursing, health, or public policy

The results made it possible to identify weaknesses in patient safety culture during the COVID-19 pandemic and may lead to the creation of strategies and interventions aimed at improving work processes and patient healthcare and safety.

## CONCLUSIONS

The study identified that all dimensions of patient safety culture in a tertiary hospital need to be improved, especially those relating to “Nonpunitive response to error” and “Staffing”. With the pandemic, these weaknesses became even more evident, requiring attention and incisive interventions from the institution’s leaders. According to data from this study, closed sectors showed a stronger safety culture compared to open sectors. Indirect patient care sectors had a low general perception of patient safety when compared to direct care sectors, which may be an interfering factor in the organization’s high reliability.

It is possible to conclude that the hospital investigated presents a weak scenario in relation to patient safety culture. Therefore, it is necessary for leaders to recognize the dimensions identified as fragile and to focus on work processes on the relevance of adopting strategies to promote a culture of patient safety shared in hospital sectors.

## Data Availability

https://doi.org/10.48331/scielodata.DGI70P
