# Supplementary material for: Patient safety culture in times of the COVID-19 pandemic: a cross-sectional study in a hospital
Source: Rev Bras Enferm. 2024 Aug 30;77(Suppl 1):e20230187. doi: 10.1590/0034-7167-2023-0187 (PMC11368380; doi:10.1590/0034-7167-2023-0187)
Supplement: Translation

# Translation: Cultura de segurança do paciente em tempos de pandemia de COVID-19: estudo transversal em hospital

**DOI:** 10.1590/0034-7167-2023-0187pt

**Authors:** Amanda Lobato Lopes, Rosana Aparecida Pereira, Laura Martins Valdevite Pereira, Felippe Micheli Costa de Castilho, Fernanda Raphael Escobar Gimenes

**Affiliations:** I Universidade de São Paulo. Ribeirão Preto, São Paulo, Brasil

**Keywords:** Segurança do Paciente, Qualidade da Assistência à Saúde, Cultura Organizacional, Hospital, COVID-19

## Abstract

**Objetivos:**

avaliar a cultura de segurança do paciente no contexto da pandemia de COVID-19 e identificar as dimensões que precisam ser aprimoradas no ambiente hospitalar e qual setor, aberto ou fechado, de assistência direta ou indireta, exibe um nível mais elevado de cultura de segurança.

**Métodos:**

estudo descritivo e transversal. Aplicou-se a versão validada para o Brasil do instrumento *Hospital Survey on Patient Safety Culture* para avaliar a cultura de segurança do paciente. Foram consideradas dimensões fortalecidas aquelas com 75% de respostas positivas.

**Resultados:**

todas as dimensões apresentaram resultados menores que 75% de respostas positivas. Setores fechados mostraram cultura de segurança mais fortalecida em relação aos abertos. Setores de assistência indireta apresentaram baixa percepção geral de segurança do paciente, quando comparados aos de assistência direta.

**Conclusões:**

com a pandemia, os pontos de fragilidade tornaram-se ainda mais evidentes, exigindo atenção e intervenções incisivas por parte das lideranças da instituição.

## INTRODUÇÃO

Em 31 de dezembro de 2019, o *Wuhan Municipal Health Commission* divulgou, em seu *website,* a ocorrência de casos de pneumonia atípica na República Popular da China^(1)^. Em 09 de janeiro de 2020, as autoridades chinesas determinaram que o surto da doença era causado por um novo coronavírus, o SARS-CoV-2, publicamente conhecido como COVID-19, infecção responsável por uma síndrome respiratória potencialmente fatal^(1)^.

Devido à rápida disseminação da doença para diversos países, em 11 de fevereiro de 2020, a Organização Mundial da Saúde (OMS) caracterizou o surto da doença como pandemia^(2)^. Entre 01 de janeiro de 2020 e 18 de maio de 2021, foram confirmados, no mundo, 163.312.429 casos e 3.386.825 mortes^(3)^. No Brasil, o primeiro caso da doença ocorreu na cidade de São Paulo no dia 26 de fevereiro de 2020^(4)^. Na cidade de Ribeirão Preto, estado de São Paulo, no ano de 2020, foram confirmados 41.977 casos e 1.045 óbitos^(5)^; no ano de 2021, foram 73.272 casos e 1.995 óbitos; e de 01 de janeiro a 16 de agosto de 2022, foram 53.402 casos e 368 óbitos^(6)^.

Manejar uma pandemia requer estrutura hospitalar robusta que proporcione tomada de decisão rápida e assertiva para o controle e a propagação do vírus^(7)^. Nessa perspectiva, a pandemia de COVID-19 desafiou os Sistemas de Assistência à Saúde (SAS) em escala global, e, no Brasil, esse cenário exigiu que o Sistema Único de Saúde (SUS) também se adaptasse para o enfrentamento da crise^(8)^.

Diante desse cenário adverso, rápidas mudanças nos modelos de prestação de cuidados foram necessárias, incluindo aumento da carga de trabalho, redistribuição de pessoal para ambientes clínicos desconhecidos e necessidade de tratar pacientes com uma nova doença e ainda pouco conhecida. Ademais, os profissionais de saúde foram incitados a desenvolver a capacidade de lidar com a doença, adaptando-se rapidamente às mudanças, superando os desafios e resistindo à pressão^(9)^.

Pesquisa prévia revelou que mudanças nas rotinas organizacionais, escassez de recursos humanos qualificados, fadiga das equipes de saúde e instituições sobrecarregadas e sob extrema pressão são fatores que contribuem para o cuidado inseguro, além de afetarem negativamente a satisfação no trabalho^(10)^. Desse modo, investigar o impacto de tais fatores na segurança do paciente, especialmente em tempos de crise sanitária, é necessário.

Segurança do paciente foi definida pela OMS (2021)^(11)^ como “estrutura de atividades organizadas que cria culturas, processos, procedimentos, comportamentos, tecnologias e ambientes de saúde que, de forma consistente e de maneira sustentável: reduz riscos e a ocorrência de dano evitável, torna o erro menos provável e reduz o seu impacto quando ocorre”.

A OMS também destacou a necessidade de serem criados sistemas de alta confiabilidade para garantir o acesso das pessoas a serviços de saúde de qualidade e seguros, por meio do desenvolvimento e da manutenção de uma cultura de segurança transparente que promova o aprendizado contínuo e que não seja punitiva^(11)^.

O termo “cultura de segurança” foi utilizado pela primeira vez na literatura pelo Grupo Consultivo Internacional em Segurança Nuclear em seu relatório sobre o acidente em Chernobyl, ocorrido em 1986^(12)^. A partir de então, o termo é utilizado por diversas instituições consideradas de alto risco, como as hospitalares^(13)^. É definida como o produto de valores, atitudes, percepções e competências, grupais e individuais, que determinam um padrão de comportamento e de comprometimento com o gerenciamento da segurança^(12)^.

Nas instituições de saúde, a cultura de segurança é definida como a associação das ações individuais e grupais que visam à redução da ocorrência de danos ao paciente como resultado das interações, atitudes e percepções sobre as questões de segurança. Estudos mostraram que existe associação direta entre a implantação da cultura de segurança nas instituições de saúde e a diminuição de eventos adversos graves e fatais^(14-15)^.

Diversos fatores interferem na cultura de segurança dos serviços de saúde, incluindo falhas na comunicação entre equipes, altas cargas de trabalho, existência de cultura punitiva e hierarquização da liderança^(10,16-17)^. Logo, para que sejam obtidas informações sobre o estado de segurança do paciente em uma instituição de saúde, investigações voltadas para a avaliação da cultura são imprescindíveis.

Há disponíveis na literatura vários instrumentos voltados para a avaliação da cultura de segurança. No presente estudo, foi utilizado o instrumento desenvolvido pela *Agency for Healthcare Research and Quality* (AHRQ) denominado *Hospital Survey on Patient Safety Culture* (HSOPSC)^(18)^, o qual foi traduzido e validado para a cultura brasileira^(19)^. O objetivo do instrumento é avaliar as múltiplas dimensões da cultura de segurança do paciente no serviço de saúde.

O HSOPSC foi utilizado em diversas pesquisas internacionais^(20-24)^. Em investigação conduzida em três hospitais públicos do Kuwait, foi realizada avaliação psicométrica do HSOPSC. Dos 22 itens relacionados ao clima de segurança, todos apresentaram carga forte entre os fatores (0,42-0,86). Ademais, em relação à análise de confiabilidade, os resultados foram satisfatórios (α> 0,60)^(22)^.

Pesquisadoras brasileiras realizaram revisão sistemática nas bases de dados PubMed, *Web of Science* e Scopus com o objetivo de identificar estudos que utilizaram o HSOPSC para coletar dados sobre a cultura de segurança e a sua contribuição para a melhoria da qualidade e segurança na assistência hospitalar^(25)^. Foram selecionados 33 estudos, conduzidos em 21 países. Com relação às dimensões do trabalho em equipe, os resultados apontaram carga forte, e as dimensões com carga fraca foram relacionadas à punição de erros relacionados ao cuidado ao paciente. Concluíram que a cultura organizacional foi fraca nas instituições avaliadas, impactando negativamente a segurança do paciente e os resultados em saúde^(25)^.

O HSOPSC é autoadministrado e avalia 12 dimensões da cultura de segurança do paciente, sendo duas relacionadas aos resultados em segurança do paciente (1 - Frequência de eventos relatados; e 2 - Percepção geral da segurança do paciente) e dez relacionadas à cultura de segurança (1 - Expectativas e ações dos supervisores/chefias de promoção de segurança; 2 - Aprendizado organizacional/melhoria contínua; 3 - Trabalho em equipe dentro das unidades; 4 - Abertura da comunicação; 5 - *Feedback* e comunicação sobre os erros; 6 - Resposta não punitiva aos erros; 7 - Quadro de profissionais; 8 - Apoio da gestão para segurança do paciente; 9 - Trabalho em equipe entre as unidades; e 10 - Passagens de plantão e transferências internas)^(19)^.

O escore é calculado utilizando uma escala do tipo Likert de cinco pontos, atribuindo valor numérico às respostas dadas pelos participantes do estudo. As respostas possuem categorias que variam de 1 – concordância (discordo totalmente; discordo; nem concordo nem discordo; concordo; e concordo totalmente) a 2 – frequência (nunca; quase nunca; às vezes; quase sempre; e sempre).

Com base no exposto, este estudo foi delineado para responder às seguintes perguntas: qual a percepção dos profissionais atuantes em hospital sobre a cultura de segurança do paciente em tempos de pandemia de COVID-19? Quais dimensões da cultura de segurança do paciente necessitam ser aprimoradas? Qual o tipo de setor (aberto e fechado) e o tipo de assistência (direta e indireta) apresentam a cultura de segurança ao paciente mais fortalecida?

## OBJETIVOS

Avaliar a cultura de segurança do paciente no contexto da pandemia de COVID-19 e identificar as dimensões que precisam ser aprimoradas no ambiente hospitalar e qual setor, aberto ou fechado, assim como entre aqueles que oferecem assistência direta e indireta, exibe um nível mais elevado de cultura de segurança.

## MÉTODOS

### Aspectos éticos

O estudo foi aprovado pelo Comitê de Ética em Pesquisa (CEP) da Escola de Enfermagem de Ribeirão Preto da Universidade de São Paulo, conforme Resolução do Conselho Nacional de Saúde nº 466/2012 do Conselho Nacional de Ética em Pesquisa do Ministério da Saúde, que aborda a ética em pesquisa com seres humanos. O Termo de Consentimento Livre e esclarecido (TCLE) foi enviado via correio eletrônico aos participantes do estudo, os quais foram informados que os resultados da pesquisa seriam destinados à publicação e que seriam garantidos o sigilo e o anonimato.

### Desenho, período e local do estudo

Trata-se de estudo descritivo e transversal, com abordagem quantitativa. Utilizou-se a lista de verificação de redação científica denominada *STrengthening the Reporting of OBservational studies in Epidemiology* (STROBE)^(26)^. O estudo foi realizado em hospital terciário de grande porte, centro de alta complexidade do interior do estado de São Paulo. O hospital é composto por três unidades, a saber: *campus;* unidade de emergência; e hospital da criança. Os dados foram coletados entre 30 de novembro de 2021 e 30 de setembro de 2022 em todos os setores das três unidades.

### População de estudo

A população-alvo do estudo foi constituída por todos os profissionais administrativos de nível superior e médio, os profissionais de saúde de nível superior e médio e os demais profissionais de nível médio e fundamental. A amostra adotada neste estudo foi de amostragem aleatória simples.

### Critérios de inclusão e exclusão

Foram incluídos os profissionais que estavam atuando há mais de três meses no hospital e que apresentavam regime de trabalho de, no mínimo, 20 horas semanais. Foram excluídos do estudo os profissionais que, durante a coleta de dados, estavam de férias ou licença e os profissionais eventuais, sem vínculo empregatício com o hospital, tais como estagiários das profissões da saúde, acadêmicos e residentes. Também foram excluídos os formulários que não foram preenchidos completamente.

### Protocolo do estudo

#### Coleta de dados

Os dados foram coletados por meio da aplicação do HSOPSC. Para assegurar a qualidade e a confiabilidade dos dados coletados, foram utilizadas as diretrizes propostas pela AHRQ^(27)^, com a validação e a inclusão de todos os profissionais que atuam nos hospitais.

Além disso, foram coletadas informações sobre os participantes da pesquisa, incluindo sexo, nível de instrução, cargo/função, tempo de serviço no hospital, carga horária semanal e interação direta com os pacientes.

A coleta de dados foi realizada de forma mista: (1) por meio da disponibilização do *link* de acesso ao instrumento eletrônico, o qual foi elaborado na plataforma virtual RedCap; e (2) presencialmente, sendo o formulário impresso e distribuído para os participantes do estudo atuantes nas três unidades hospitalares.

O *link* foi disponibilizado no período de 30 de novembro de 2021 a 7 de fevereiro de 2022, enviado por e-mail institucional, com o auxílio do Serviço de Gerenciamento de Risco. Contudo, devido à baixa adesão do público-alvo, a coleta passou a ser realizada de forma presencial por meio da distribuição de formulários impressos do período de 21 de fevereiro de 2022 a 30 de setembro do mesmo ano. Destaca-se que tal mudança foi previamente autorizada pela direção da instituição. Todos os andares das três unidades hospitalares foram visitados pela pesquisadora, a qual realizou contato prévio com o responsável pelo setor. Aos que aceitaram participar da pesquisa, foi enviado o TCLE por meio do correio eletrônico e concedido prazo de uma semana para o preenchimento. Posteriormente, os instrumentos impressos foram recolhidos pela pesquisadora, e os dados foram digitados no sistema RedCap.

#### Análise dos resultados e estatística

Os dados foram transferidos da plataforma RedCap para planilhas do programa *Microsoft Excel*® 2016. Todas as análises foram realizadas no programa *R* (R Core Team, 2021), versão 4.1.2, sendo calculados os percentuais de respostas positivas referentes às dimensões de cultura de segurança do paciente. Foram consideradas respostas positivas as alternativas 4 ou 5 (concordo/concordo totalmente ou quase sempre/sempre) em perguntas formuladas positivamente e as alternativas 1 ou 2 (discordo totalmente/discordo ou nunca/raramente) em perguntas formuladas negativamente. Com relação às respostas negativas, foram consideradas as alternativas 1 ou 2 para perguntas formuladas positivamente e 3 ou 4 para perguntas formuladas negativamente. Foram consideradas respostas neutras aquelas registradas como “nem discordo nem concordo” ou “às vezes”, conforme proposto pela AHQR^(27)^.

Foram consideradas áreas fortes as dimensões referentes à cultura de segurança do paciente, que obtiveram 75% de respostas positivas. Foram consideradas áreas frágeis e que necessitam de melhoria as dimensões que obtiveram 50% ou menos de respostas positivas^(27)^.

A comparação entre os setores abertos e fechados foi realizada segundo a classificação proposta por Bianchi^(28)^, levando-se em consideração o fluxo de pacientes e familiares. Foram considerados setores abertos os ambulatórios, setor de oncologia, Unidade de Transplante de Fígado, Unidade de Transplante Renal, além das enfermarias de psiquiatria, clínica médica, hematologia, ginecologia e obstetrícia, cirurgia, ortopedia, oftalmologia, otorrino e pediatria. Os setores fechados foram administração, contabilidade, recursos humanos, Núcleo Interno de Regulação (NIR), Centro Integrado da Qualidade, nutrição e dietética, serviço de distribuição, central de materiais, unidade respiratória, laboratórios de microbiologia, Centro de Reabilitação (CER), unidade de diálise, banco de sangue, agência transfusional, controle de risco, farmácia, centro cirúrgico, recuperação pós-anestésica, Centro de Terapia Intensiva (CTI) adulto, pediátrico e neonatal, neurologia, Unidade Coronariana (UCO), unidade de queimados, central de quimioterapia, Unidade de Tratamento de Doenças Infecciosas (UTDI), semi-intensivo de clínica médica e Centro de Cirurgia de Epilepsia (CIREP).

Também foi realizada a comparação entre setores de assistência direta e indireta, seguindo como referencial teórico a classificação de Bartolomei^(29)^, que distingue a assistência de saúde como direta, sendo a assistência oferecida com relação direta com o usuário, e como indireta, sendo atos que configuram conforto e segurança física e material ao paciente. Para assistência indireta, foram considerados todos os profissionais que trabalham em setores de gestão e administração, distribuição de materiais, farmácia, nutrição e laboratórios. Os profissionais de todos os ambulatórios, enfermarias, CTIs, centro cirúrgico, unidades de tratamento, como diálise, queimados, reabilitação e outros, foram considerados de assistência direta. Foi utilizado o teste não paramétrico Kruskal-Wallis, uma escolha adequada para a comparação de três ou mais amostras independentes. O nível de significância adotado neste estudo foi estabelecido em 95%.

## RESULTADOS

Foram preenchidos 459 formulários, dos quais 111 (24,2%) foram preenchidos no formato *online,* enquanto que 348 (75,8%) foram preenchidos no formato impresso. Com relação à unidade de trabalho dos profissionais, 327 (71,2%) atuavam na unidade do *campus;* 89 (19,4%) atuavam na unidade de emergência; e 43 (9,4%) atuavam no hospital infantil. Com relação ao tipo de setor, foram 139 (30,3%) participantes de unidades abertas e 320 (69,7%) de unidades fechadas. Considerando-se o tipo de assistência, 388 (84,5%) prestavam assistência direta ao paciente, e 71 (15,5%), assistência indireta. Entre os participantes, a maioria era do sexo feminino (367; 80,1%) e com idade média de 43,53 anos (21,07 ± 70,22). A Tabela 1 apresenta a caracterização dos participantes.

**Tabela 1:** Caracterização dos profissionais que atuam nas três unidades hospitalares participantes da pesquisa (N=459); Ribeirão Preto, São Paulo, Brasil, 2022

Variáveis	Frequência n (%)
Sexo	
Feminino	367 (80,1)
Masculino	91 (19,9)
Grau de instrução	
Ensino médio ou menos	134 (29,2)
Superior	142 (31)
Especialização	115 (25,1)
Mestrado ou doutorado	68 (14,8)
Cargo/função	
Auxiliares/técnicos*	248 (54,0)
Graduados**	211 (46,0)
Tempo de trabalho no hospital	
≤ 5 anos	161 (35,1)
6 a 10 anos	86 (18,7)
11 a 15 anos	53 (11,5)
16 a 20 anos	27 (5,9)
>20 anos	132 (28,8)
Carga horária semanal	
< 40 horas	264 (57,5)
≥ 40 horas	295 (42,5)
Contato direto com o paciente	
Sim	336 (73,2)
Não	123 (26,8)

* Auxiliares/técnicos: auxiliar e técnico de enfermagem; demais profissionais de nível técnico, como aqueles atuantes em eletrocardiograma, laboratório, radiologia e farmácia; auxiliar administrativo/secretário; outro; **Graduados: médico do corpo clínico/médico assistente; enfermeiro; farmacêutico/bioquímico/biólogo/biomédico;nutricionista; fisioterapeuta, terapeuta respiratório, terapeuta ocupacional ou fonoaudiólogo; psicólogo; assistente social; administração/direção.

Considerando as 42 questões do HSOPSC, que foram agrupadas em 12 dimensões da cultura de segurança, foi calculado o percentual de respostas positivas, neutras e negativas, conforme Figura 1.

**Figura 1:** Distribuição de respostas negativas, neutras e positivas para as 12 dimensões de segurança avaliadas por meio do Hospital Survey on Patient Safety Culture, Ribeirão Preto, São Paulo, Brasil, 2022

Nenhuma das 12 dimensões avaliadas atingiu o percentual de 75% de respostas positivas. Apesar disso, destacam-se as dimensões que expressaram maiores percentuais de respostas positivas, ou seja, 67% em “Expectativas e ações dos supervisores/chefias de promoção de segurança” e 62,7% em “Trabalho em equipe dentro das unidades”.

Ainda, sete das 12 dimensões obtiveram 50% ou menos de respostas positivas, representando áreas frágeis de segurança do paciente e que necessitam de melhoria, sendo elas, em ordem decrescente: “*Feedback* e comunicação sobre os erros” (49,4%); “Percepção geral da segurança do paciente” (44,8%); “Apoio da gestão para segurança do paciente” (44%); “Trabalho em equipe entre as unidades” (36,4%); e “Passagens de plantão e transferências internas” (35,7%). As duas dimensões com os menores percentuais de respostas positivas se destacaram: “Quadro de profissionais” (33,5%); e “Resposta não punitiva aos erros” (18,6%).

Em relação à variável “eventos adversos notificados pelos profissionais nos últimos 12 meses”, a Figura 2 evidenciou que a maioria (67,5%) não realizou nenhuma notificação.

**Figura 2:** Eventos adversos notificados pelos profissionais nos últimos 12 meses (N = 459), Ribeirão Preto, São Paulo, Brasil, 2022

No que diz respeito ao grau geral de segurança do paciente, os resultados revelaram que, dos 459 (100%) participantes, 218 (47,5%) consideram a segurança do paciente do hospital muito boa.

No presente estudo, foi comparada a porcentagem de respostas positivas para a segurança do paciente entre setores fechados e abertos. A Tabela 2 evidencia que os setores fechados obtiveram maiores porcentagens de respostas positivas em todas as 12 dimensões de segurança do paciente, em comparação com os setores abertos; contudo, os resultados não foram significativos, exceto para “Expectativas e ações dos supervisores/chefias de promoção de segurança” (p-0,015), “*Feedback* e comunicação sobre os erros” (p-0,0077), “Abertura da comunicação” (p-0,0156) e “Trabalho em equipe entre as unidades” (p-0,0039).

**Tabela 2:** Distribuição de respostas positivas para as 12 dimensões de segurança do paciente avaliadas por meio do Hospital Survey on Patient Safety Culture de acordo com o tipo de setor, Ribeirão Preto, São Paulo, Brasil, 2022

Dimensões da segurança do paciente	Tipo de setor (%)		Valor de p*
	Aberto	Fechado	
1 - Trabalho em equipe dentro das unidades	62,2	62,7	0,7481
2 - Expectativas e ações dos supervisores/chefias de promoção de segurança	65,7	67,5	0,015
3 - Aprendizado organizacional/melhoria contínua	51,1	59,5	0,0996
4 - Apoio da gestão para segurança do paciente	40	45,7	0,1464
5 - Percepção geral da segurança do paciente	41,4	46,3	0,5897
6 - *Feedback* e comunicação sobre os erros	48,6	49,1	0,0077
7 - Abertura da comunicação	55,6	55,7	0,0156
8 - Frequência de eventos relatados	53	54,4	0,6029
9 - Trabalho em equipe entre as unidades	31,9	39,2	0,0039
10 - Quadro de profissionais	28,4	35,8	0,3583
11 - Passagens de plantão e transferências internas	34,2	36,4	0,843
12 - Resposta não punitiva aos erros	14,6	20,3	0,4166

* *Kruskal-Wallis.*

Também foi realizada a comparação da cultura de segurança do paciente entre setores que prestam assistência direta ao paciente e setores que prestam assistência indireta. A Tabela 3 revela que, nos setores onde é prestada assistência indireta aos pacientes, a porcentagem de respostas positivas foi maior (oito das 12 dimensões) do que quando comparada a de setores que prestam assistência direta. Contudo, os resultados não foram significativos, exceto para o domínio “Aprendizado organizacional/melhoria contínua”, com valor final aferido em p=0,0033.

**Tabela 3:** Distribuição de respostas positivas para as 12 dimensões de segurança avaliadas por meio do Hospital Survey on Patient Safety Culture de acordo com o tipo de assistência prestada aos pacientes, Ribeirão Preto, São Paulo, Brasil, 2022

Dimensões da segurança do paciente	Tipo de Assistência (%)		Valor de p*
	Direta	Indireta	
1 - Trabalho em equipe dentro das unidades	62,6	62,3	0,5256
2 - Expectativas e ações dos supervisores/chefias de promoção de segurança	65,7	74	0,2086
3 - Aprendizado organizacional/melhoria contínua	56	62	0,0033
4 - Apoio da gestão para segurança do paciente	42,3	53,5	0,497
5 - Percepção geral da segurança do paciente	45,2	43	0,1183
6 - *Feedback* e comunicação sobre os erros	48,6	58,3	0,8998
7 - Abertura da comunicação	55,7	63,8	0,7259
8 - Frequência de eventos relatados	53,9	54,9	0,8569
9 - Trabalho em equipe entre as unidades	37,5	47,5	0,074
10 - Quadro de profissionais	34	31,1	0,0589
11 - Passagens de plantão e transferências internas	35,8	33,8	0,3574
12 - Resposta não punitiva aos erros	18,2	20,7	0,4737

* *Kruskal-Wallis.*

## DISCUSSÃO

O objetivo geral do estudo foi avaliar a cultura de segurança do paciente no contexto da pandemia de COVID-19 e identificar as dimensões que precisam ser aprimoradas no hospital. Os resultados mostraram que nenhuma das 12 dimensões de segurança avaliadas por meio do HSOPSC foram consideradas áreas fortes, pois não apresentaram porcentagem ≥75% de respostas positivas.

Esse mesmo cenário foi apresentado em revisão de escopo que reuniu vários estudos desenvolvidos no Brasil. De acordo com os pesquisadores, a maioria dos hospitais brasileiros não apresenta nenhuma dimensão forte^(30)^.

Contudo, no presente estudo, 47,5% dos participantes consideram a segurança do paciente do hospital “muito boa”. Algumas dimensões apresentaram potencial de se tornar fortalecidas, tais como “Expectativas e ações dos supervisores/chefias de promoção de segurança” e “Trabalho em equipe dentro das unidades”. Esse resultado corrobora o de pesquisa conduzida em hospital filantrópico de Diamantina, estado de Minas Gerais^(31)^. Os resultados demonstram a importância de as lideranças manifestarem interesse na temática, incentivando boas práticas, investindo em processos seguros de trabalho e elogiando ações executadas com excelência, assim motivando os colaboradores a melhorar a segurança dos pacientes. Quando os profissionais alcançam essa visão, o trabalho em equipe torna-se uma realidade, como ilustra esta pesquisa, em que o apoio e o respeito entre as pessoas são presentes, favorecendo, assim, uma assistência segura e de qualidade.

Em revisão sistemática realizada em 21 países, em sua maioria europeus e asiáticos^(25)^, foi demonstrado que mesmo considerando a diversidade de cada um, as dimensões que aparecem em destaque com menores escores de respostas positivas foram “Resposta não punitiva aos erros” e “Quadro de profissionais” representando as áreas mais fragilizadas, bem como no presente estudo, que mostrou os percentuais de 18,6% e 33,5%, respectivamente.

No contexto de segurança do paciente, a “Resposta não punitiva aos erros” expõe a cultura de culpabilidade que ainda se faz presente em muitas instituições hospitalares, onde os profissionais sentem apreensão em relatar erros devido ao possível medo de serem responsabilizados e, consequentemente, punidos, conduzindo à subnotificação de eventos^(32)^. Esse ponto pode ser confirmado pelo percentual de notificações nos 12 meses anteriores à pesquisa, em que mais da metade (67,5%) dos profissionais não realizou nenhuma notificação. Outras pesquisas que utilizaram o mesmo instrumento obtiveram resultados semelhantes para essas dimensões^(30, 32, 33)^.

Essa conjuntura de subnotificação de eventos adversos é agravada pela dimensão de “Quadro de profissionais”, destacada como a segunda área mais fragilizada do estudo. Somando-se ao cenário adverso de pandemia de COVID-19, traz o aumento da carga de trabalho, redistribuição de pessoal para ambientes clínicos desconhecidos e necessidade de tratar pacientes com uma nova doença, acarretando no aumento do estresse do profissional^(9)^, fatores esses que podem refletir na subnotificação.

Ao relacionar as áreas fragilizadas do hospital investigado no contexto da pandemia a estudos anteriores conduzidos em outros cenários e contextos, verifica-se que a pandemia ressaltou pontos de fragilidade que já eram considerados desafiadores, incluindo a cultura de culpabilização e punição aos erros, expressa nesses estudos pela menor porcentagem de respostas positivas para as dimensões “Resposta não punitiva aos erros” e “Quadro de profissionais”, relacionadas com a sobrecarga de trabalho e inadequação de recursos humanos^(30, 32, 33)^.

No presente estudo, os setores abertos apresentaram menor porcentagem de respostas positivas em todas as dimensões de segurança do paciente, quando comparados aos setores fechados. Como não foram encontrados estudos anteriores que avaliam as dimensões entre setores abertos e fechados, não foi possível essa comparação.

No que diz respeito aos setores de assistência direta e indireta, os setores de assistência indireta, que incluem predominantemente a gestão e a administração, obtiveram maior porcentagem de respostas positivas para a maioria das dimensões. Estudo realizado nos EUA como uma extensão do HSOPSC evidenciou que setores de gestão tendem a acreditar que a cultura de segurança do paciente na instituição seja mais forte do que realmente é^(34)^. Esse fato, no presente estudo, pode ter influenciado a maior positividade das dimensões de segurança do paciente em setores indiretos.

Em contrapartida, na dimensão “Percepção geral da segurança do paciente”, setores de assistência indireta obtiveram porcentagem de resposta positiva menor (43%) que os setores de assistência direta (45,2%), evidenciando possível dificuldade em avaliar a cultura de segurança na instituição. Relacionado a esse contexto, destaca-se um dos principais desafios da coleta de dados do presente estudo, que seria o baixo engajamento dos profissionais de setores com assistência indireta, principalmente dos profissionais administrativos. De acordo com relatos desses profissionais, os mesmos não percebem o seu papel na segurança do paciente por não estarem diretamente ligados à assistência. Ainda em conformidade ao estudo desenvolvido nos EUA, cargos administrativos - assistência indireta - são áreas com maiores lacunas na percepção da cultura de segurança do paciente, reforçando a importância da formação e treinamento desses profissionais acerca da temática para que esses se tornem corresponsáveis pela segurança do paciente e a instituição como um todo alcance uma cultura de segurança compartilhada^(34)^.

Ademais, pesquisa realizada em Unidade de Terapia Intensiva de hospital universitário apontou que o grupo de pacientes que apresentou incidentes de segurança relacionados a falhas administrativas necessitou de maior tempo de internação e maiores cuidados pela equipe de enfermagem^(35)^. Tal cenário revela que todas as áreas profissionais envolvidas no processo da assistência, seja direta ou indireta, devem participar e se engajar nas estratégias de segurança. Tendo isso em vista, é importante que as instituições repensem a cultura de segurança vigente, de modo a se tornar uma organização de alta confiabilidade. Tais organizações apresentam uma condição de atenção plena e persistente voltada para o reconhecimento precoce dos erros e para a intervenção imediata, de modo a evitar que tais eventos se tornem catastróficos. Ademais, organizações de alta confiabilidade cultivam a resiliência ao priorizar incansavelmente a segurança em detrimento de outras medidas de desempenho^(36)^.

### Limitações do estudo

Entre as limitações do estudo, é possível citar a inexistência de pesquisas utilizando o mesmo instrumento, realizadas no contexto da pandemia de COVID-19, inviabilizando a comparação dos resultados. Além disso, observou-se a falta de adesão dos profissionais em participar da pesquisa e/ou o preenchimento inadequado do formulário.

Outra limitação é a possibilidade de viés das respostas por parte dos participantes. Dependendo da sensibilidade ou das percepções individuais, os participantes podem ser inclinados a fornecer respostas que refletem uma visão mais favorável da cultura de segurança, o que pode afetar a precisão dos resultados. Ademais, a amostra aleatória simples pode não representar completamente a diversidade de contextos hospitalares, limitando a generalização dos resultados para outras instituições de saúde.

### Contribuições para as áreas da enfermagem, saúde, ou políticas públicas

Os resultados permitiram identificar as fragilidades na cultura de segurança do paciente no contexto da pandemia de COVID-19 e poderão direcionar à criação de estratégias e intervenções voltadas para a melhoria dos processos de trabalho e da assistência em saúde e segurança do paciente.

## CONCLUSÕES

O estudo permitiu identificar que todas as dimensões da cultura de segurança do paciente de hospital de nível terciário necessitam ser aprimoradas, principalmente as que versam sobre “Resposta não punitiva aos erros” e “Quadro de profissionais”. Com a pandemia, tais fragilidades tornaram-se ainda mais evidentes, exigindo atenção e intervenções incisivas por parte das lideranças da instituição. De acordo com dados deste estudo, setores fechados mostraram cultura de segurança mais fortalecida em relação aos abertos. Setores de assistência indireta ao paciente apresentaram baixa percepção geral de segurança do paciente, quando comparados aos de assistência direta, o que pode ser um fator de interferência na alta confiabilidade da organização.

É possível concluir que o hospital investigado apresenta um cenário de fragilidade em relação à cultura de segurança do paciente. Logo, é necessário que as lideranças reconheçam as dimensões apontadas como frágeis e que coloquem em foco nos processos de trabalho a relevância de adotar estratégias para promover a cultura de segurança do paciente compartilhada nos setores do hospital.

## Data Availability

https://doi.org/10.48331/scielodata.DGI70P
